# Robust microscale superlubricity under high contact pressure enabled by graphene-coated microsphere

**DOI:** 10.1038/ncomms14029

**Published:** 2017-02-14

**Authors:** Shu-Wei Liu, Hua-Ping Wang, Qiang Xu, Tian-Bao Ma, Gui Yu, Chenhui Zhang, Dechao Geng, Zhiwei Yu, Shengguang Zhang, Wenzhong Wang, Yuan-Zhong Hu, Hui Wang, Jianbin Luo

**Affiliations:** 1 State Key Laboratory of Tribology, Tsinghua University, Beijing 100084, China; 2 Beijing National Laboratory for Molecular Sciences, Institute of Chemistry, Chinese Academy of Sciences, Beijing 100190, China; 3 School of Mechanical Engineering, Beijing Institute of Technology, Beijing 100081, China

## Abstract

Superlubricity of graphite and graphene has aroused increasing interest in recent years. Yet how to obtain a long-lasting superlubricity between graphene layers, under high applied normal load in ambient atmosphere still remains a challenge but is highly desirable. Here, we report a direct measurement of sliding friction between graphene and graphene, and graphene and hexagonal boron nitride (h-BN) under high contact pressures by employing graphene-coated microsphere (GMS) probe prepared by metal-catalyst-free chemical vapour deposition. The exceptionally low and robust friction coefficient of 0.003 is accomplished under local asperity contact pressure up to 1 GPa, at arbitrary relative surface rotation angles, which is insensitive to relative humidity up to 51% RH. This ultralow friction is attributed to the sustainable overall incommensurability due to the multi-asperity contact covered with randomly oriented graphene nanograins. This realization of microscale superlubricity can be extended to the sliding between a variety of two-dimensional (2D) layers.

## Abstract (web summary)

Superlubricity can be unstable in graphene systems, especially under high applied loads. Here the authors use microspheres uniformly coated by graphene to measure friction between 2D materials and show that superlow friction is preserved for long periods of time under high loads and various atmospheres.

Superlubricity or negligible sliding friction with exceptionally low energy dissipation is crucial for energy-savings, environmental protection and long-life machine operation in industrial applications^1^,^2^,^3^,^4^,^5^,^6^,^7^,^8^. Owing to the weak interlayer van der Waals interaction, atomically thin graphene and two-dimensional (2D) materials have stood out in the efforts of seeking new materials for further reduction in friction^9^,^10^,^11^,^12^,^13^,^14^,^15^, showing promising prospects to lubricate micro- or nano-electro mechanical systems (MEMS/NEMS). There are a few up-to-date experimental studies reporting the observation of structural lubricity of 2D materials at different length scales, such as superlubricity of graphite^16^, superlubric sliding of graphene nanoflakes on graphite^17^, ultralow friction by confining C_60_ molecular layer between graphite layers at the nanoscale^18^ and self-retracting motion of cleaved graphite mesas at the mesoscale^19^. Recently, the adhesion and superlow friction force has been directly measured in mesoscale graphite contacts by inducing a shear glide along a single basal plane of highly oriented pyrolytic graphite (HOPG) (refs 20, 21). The ultralow friction between the graphene layers has been found to be strongly dependent on the interlayer rotational alignment, which is attributed to the incommensurability between the contacting surfaces^16^. Further studies have shown the instability of this superlubricity state and the retrieval of high friction state during the sliding process due to the torque-induced reorientation and a transition from incommensurate to commensurate contact^22^.

Recently, stable macroscopic superlubricity has also been realized in a dry nitrogen environment between diamond-like carbon and graphene films with the formation of graphene nanoscrolls, originating from the sliding induced spontaneous wrapping of graphene flakes around nanodiamond particles. And the sliding happens between the graphene nanoscrolls and randomly arranged diamond-like carbon atoms, forming an incommensurate contact^23^. Another example of macroscale superlubricity is demonstrated between the inner and outer shells of centimeters-long double-walled carbon nanotubes^24^. Overall, the realization of a sustained incommensurate sliding contact is the main focus to achieve stable superlubricity. It's still a big challenge, however, to achieve superlubricity under high applied normal loads, in various atmospheres, and at arbitrary interlayer relative rotation angles, which is important for real applications of graphene and 2D materials.

In this paper, we conceive a method to directly measure the friction between 2D materials under high applied normal loads by using graphene-coated microsphere (GMS). Graphene-coated commercial atomic force microscopy (AFM) sharp tip has already been developed for studies of electronic properties of materials^25^,^26^. However, a more conformal graphene/graphene contact condition is required in order to measure the friction between graphene layers. Hence we use GMS probe (8 μm in diameter) instead of single-asperity tip under the applied load as high as 1.45 μN. The key challenge is to transfer or directly fabricate graphene film homogeneously on the microsphere probe, especially on insulating materials. Here, a metal-catalyst-free chemical vapour deposition (CVD) method is employed to grow polycrystalline graphene directly on SiO_2_ microsphere^27^. This GMS probe shows excellent friction reduction performance when sliding on different substrates, especially on clean single crystalline graphite surfaces, where a state of microscale superlubricity is achieved.

## Results

### Preparation and characterization of GMS microsphere probe

**Figure 1:**
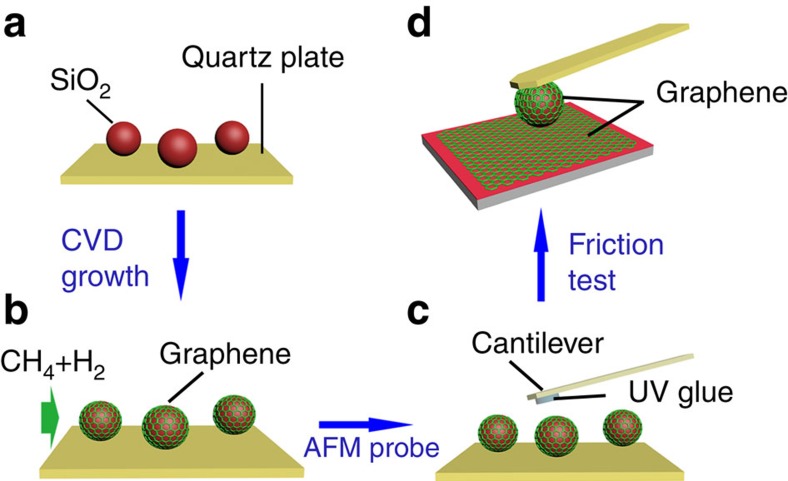
Schematic fabrication process of graphene-coated SiO_2_ microsphere probe. (**a**) Step 1: SiO_2_ microspheres dispersed on quartz plate. (**b**) Step 2: CVD growth of multi-layer graphene film on SiO_2_ microsphere. (**c**) Step 3: Graphene-coated SiO_2_ microsphere attached to AFM cantilever with UV light solidify glue. (**d**) Step 4: Tribological test of graphene-coated microsphere probe by friction force microscopy.

The graphene film was grown directly on the 8 μm SiO_2_ microspheres by an atmospheric CVD system. Schematic illustration of the preparation process is shown in Fig. 1. SiO_2_ microspheres were firstly dispersed in ethanol, and then transferred onto quartz plate by capillary glass tube. The quartz plate together with the SiO_2_ microspheres were placed at the centre of quartz tube in a CVD furnace. The CVD growth procedure involves pre-heating, substrate annealing and high-temperature growth in a gas atmosphere consisting of methane, hydrogen and argon. This method avoids the participation of metal catalyst and complicated post-growth transfer processes. The detailed growth processes are described in the Methods section. After the CVD growth process, the graphene-coated SiO_2_ microsphere was attached to a tipless silicon cantilever with ultraviolet (UV) light solidify glue to yield a GMS probe.

**Figure 2:**
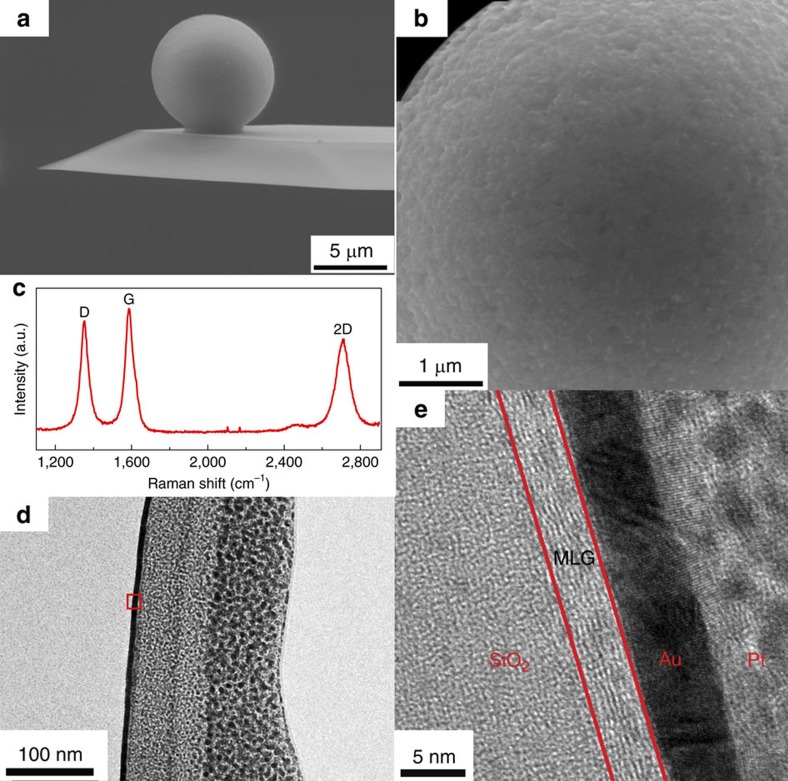
Structural characterization of multilayer graphene (MLG) coated SiO_2_ microsphere. (**a**) SEM side view of the graphene-coated microsphere probe. (**b**) SEM top view of the microsphere. (**c**) Raman spectra of the MLG on the microsphere. (**d**) TEM images of MLG coated on the SiO_2_ microsphere. (**e**) Zoom in view of the red square marked in **d**. Pt and Au films are deposited as the protective film during TEM sample preparation of focused ion beam (FIB) process.

Figure 2a,b shows the scanning electron microscopy (SEM) images of the graphene-coated SiO_2_ microsphere. The microsphere surface is covered with a continuous and homogeneous graphene film. The morphology of the graphene film is relatively rough, ascribing to the original rough surface of the bare SiO_2_ microsphere substrate. The existence of graphene film on SiO_2_ microsphere is firstly verified by Raman spectroscopy, as shown in Fig. 2c, which demonstrates the presence of G, 2D and D peaks at about 1,580, 2,700 and 1,350 cm^−1^, respectively. The ratio of the relative intensity between 2D and G peak (I_G_/I_2D_) indicates a multilayer graphene (MLG) structure on the microsphere^28^. Moreover, the D peak observed at 1,350 cm^−1^ shows structural disorder or effects of grain boundaries. An average grain size of approximately 160 nm is estimated by atomically resolved AFM measurement for graphene films grown on flat SiO_2_ substrate (see Supplementary Figs 1–5 and Supplementary Note 1). Furthermore, the growth of graphene on curved and rough SiO_2_ microsphere surface can lead to an increase in the number of nucleation sites of graphene, and a further decrease in the grain size as compared to that on the flat SiO_2_/Si substrate^29^. As a consequence, it is reasonable to deduce that under the same CVD process, the average grain size should be no more than 160 nm for the GMS probe.

Cross-sectional high resolution transmission electron microscope (HRTEM) was employed in order to further investigate the structure and thickness of the graphene film on the microsphere. Au and Pt films were firstly sputtered on the region of interest to prevent the graphene film from further damage during the TEM specimen preparation. The sample was then picked out from the top of the microsphere. The four different layers of the HRTEM image in Fig. 2e from left to right are SiO_2_ substrate, MLG, Au and Pt film, respectively. The HRTEM image shows unambiguous evidence of the growth of a homogeneous MLG film on the insulating SiO_2_ microsphere with lamellar structure. The thickness of the graphene film is about 8–10 atomic layers, stacking in the surface normal direction. The coverage of graphene (0001) basal plane on the microsphere is assumed to be beneficial to reduce sliding friction due to the low shear strength between graphene layers.

### Frictional characteristics of graphene-coated microsphere

**Figure 3:**
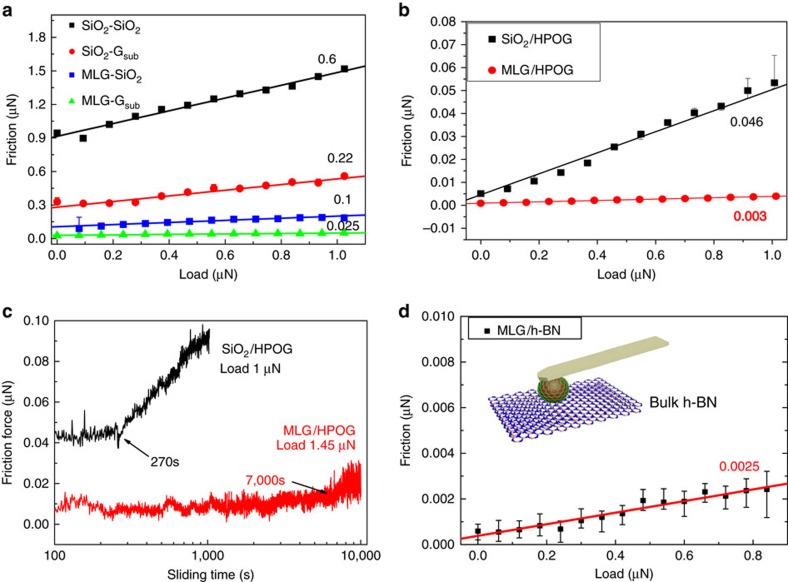
Tribological behaviours of the graphene-coated microsphere. (**a**) Friction force as a function of the applied normal load for different combinations of sliding materials: SiO_2_ microsphere sliding against SiO_2_ substrate (SiO_2_/SiO_2_), SiO_2_ microsphere against transferred CVD grown graphene on SiO_2_ substrate (SiO_2_/G_sub_), multilayer graphene-coated SiO_2_ microsphere against SiO_2_ substrate (MLG/SiO_2_) and MLG-coated microsphere against transferred CVD grown graphene on substrate (MLG/G_sub_). (**b**) Friction force as a function of the applied normal load for SiO_2_ microsphere sliding against HOPG (SiO_2_/HOPG) and MLG-coated SiO_2_ microsphere against HOPG (MLG/HOPG), respectively. The straight line represents the line fitting of collected data and the error bars correspond to a standard deviation in these measurements. The slope of each fitting line (defined as the friction coefficient) is denoted. (**c**) Time evolution of friction force for MLG/HOPG (applied load 1.45 μN) and SiO_2_/HOPG (applied load 1 μN). (**d**) Friction force as a function of load for MLG/h-BN tribo-pair. Inset picture shows the schematic of the friction test. The friction coefficient is 0.0025 by the slope of the fitting line.

The friction measurement was mainly conducted by MFP3D AFM in lateral force mode under ambient atmosphere, as well as by NT-MDT AFM with an environmental chamber under dry nitrogen and different humidity. Friction force was measured by the half width of the lateral force loop under different applied loads, as shown in Fig. 3a. The four groups of sliding materials therein for friction tests in ambient air are denoted as ‘microsphere surface material/substrate material': SiO_2_/SiO_2_, SiO_2_/G_sub_, MLG/SiO_2_ and MLG/G_sub_, where MLG represents multilayer graphene grown directly on SiO_2_ microsphere, and G_sub_ represents the monolayer graphene firstly grown on Pt foil by CVD process and then bubbling transferred onto the flat SiO_2_/Si substrate^30^. Obviously, the average friction force increases linearly with normal load for all the cases, according to the fitting lines in Fig. 3a,b, the slopes of which are also denoted. The linear relationship between the friction force and the normal load is similar to the typical macroscopic friction law rather than the single-asperity model, which is characteristics of rough contacts and has been predicted by a series of macroscale roughness theories, such as Greenwood and Williamson model^31^, and has been recently extended to describe the behaviours of nanoscale contacts^32^. So our results predict a multi-asperity contact rather than a single-asperity one for the 8 μm diameter GMS, which is consistent with the SEM observation of the relatively rough morphology of the microsphere in Fig. 2b. Interestingly, friction is also observed at zero or even negative applied load, indicating the effect of adhesion force on friction (see Fig. 3a,b, Supplementary Figs 6 and 7 and Supplementary Note 2) (ref. 33). From the above discussions, the friction force can be expressed in equation (1),


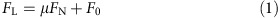


where *F*_L_ stands for the friction force, *μ* represents the slope of the friction versus normal force curve, which is defined as friction coefficient here, *F*_N_ is the applied normal force and *F*_0_ is the offset friction force when the applied load is 0. We found that even for the MLG/HOPG system, the offset force is positive (around 1∼3 nN) at zero applied load (Supplementary Fig. 7a). As can be seen in the inset of Supplementary Fig. 7a, the adhesion force for MLG/HOPG is around 71 nN, which explains the positive offset friction force. The strong influence of adhesion force on nanoscale friction laws has been studied by molecular dynamics (MD) simulations, where a transition from linear to sublinear dependence of friction force on load takes place for adhesion governed regime, yet opposite effect is predicted by increasing the roughness of the interface^32^. So the linear load dependence of friction in our experiment again confirms a rough contact between the GMS and the flat samples.

By comparing different tribo-pairs, we find that not only the absolute value of the friction force, but also the slope of the fitting line *μ* differs from one another. The friction coefficient of the bare SiO_2_ microsphere probe sliding on SiO_2_ substrate (SiO_2_/SiO_2_) is as high as 0.6. However, with the presence of monolayer graphene on the substrate (SiO_2_/G_sub_), the friction coefficient decreases markedly to 0.22. Similarly, with the growth of MLG on the SiO_2_ microsphere (MLG/SiO_2_), the friction coefficient decreases to 0.1. When both sliding surfaces are coated with graphene, that is, the GMS slides against graphene on substrate (MLG/G_sub_), where sliding happens between the graphene layers, the friction coefficient reaches as low as 0.025. This experimental setup enables the direct measurement of interlayer sliding friction between 2D materials, which shows excellent frictional performance of the graphene/graphene tribo-pair.

To further investigate the friction properties of the graphene-coated SiO_2_ microsphere probe, freshly cleaved HOPG (SPI-1 Grade, SPI Supplies) and nature graphite were also used as the mating material. The friction forces of the two different tribo-pairs: SiO_2_/HOPG and MLG/HOPG with a function of applied normal load, are compared in Fig. 3b. For SiO_2_/HOPG, the friction coefficient extracted from the slope of the fitting line is 0.046, compared to that of 0.22 for the SiO_2_/graphene tribo-pair, indicating a better frictional performance on HOPG than on CVD graphene. Two possible reasons may explain the phenomenon: Firstly, the freshly cleaved HOPG sample is usually defect-free in a relatively large single crystalline domain, as compared to the possible defects generated during the growth and transfer processes of the CVD graphene. Secondly, the bulk HOPG is atomically smooth and free from roughness and puckering effects which reportedly cause a friction rise in single- and few-layer graphene^14^. The reasons for the large friction difference between HOPG and G_sub_ substrates are further discussed by using mechanical exfoliated monolayer graphene on SiO_2_ substrate as a reference in Supplementary Note 3 and Supplementary Figs 8 and 9.

Noteworthily, the friction coefficient of MLG/HOPG tribo-pair is 0.003 calculated from the red fitting line in Fig. 3b, reaching the superlubricity regime. Atomic scale friction force curve of MLG/HOPG shows smooth sliding compared to the stick-slip fashion of SiO_2_/HOPG (Supplementary Note 4 and Supplementary Fig. 10), and the average friction force is almost vanishing from the distance between the trace and retrace profiles. The robust superlubricity is also characterized by the long-lived superlow friction coefficient under high normal load in this study. As shown in Fig. 3c, for SiO_2_/HOPG tribo-pair, the stable friction force only lasts for 270 s before failure. However, the state of superlubricity of MLG/HOPG lasts for a much longer lifetime of 7,000 s even under severer conditions, before the friction force increases slightly. After sliding for 10,000 s, the GMS probe was moved to a nearby unscratched area on HOPG, and the original superlubricity state is resumed. The microsphere after friction test shows no obvious change by Raman characterization (see Supplementary Note 5 and Supplementary Fig. 11c). However, wear track is distinguishable by AFM height image in the scan region on the graphite side after friction test (Supplementary Fig. 11b). This finding suggests the extraordinary wear resistance of graphene-coated SiO_2_ microsphere probe, and the loss of superlubricity may be attributed to the mild wear on the HOPG side.

Furthermore, we have also obtained superlow friction coefficient between GMS and graphite under different experimental conditions. As shown in Supplementary Note 6 and Supplementary Fig. 12, superlubricity can be preserved even under dry nitrogen and relative humidity of 51% as measured in the environmental chamber in NT-MDT AFM, and under scan size as large as 50 μm and scan rate as high as 100 μm s^−1^. The interesting humidity-insensitive properties of superlubricity may be particularly ascribed to the hydrophobic nature of both sides of the tribo-pair (MLG and graphite), which is verified by MD simulation in Supplementary Note 7, Supplementary Figs 13–15 and Supplementary Movies 1–6. More importantly, we have also managed to measure the sliding friction between graphene and other 2D materials like hexagonal boron nitride (h-BN), which is otherwise quite challenging without the present technique. A very low friction coefficient of 0.0025 is obtained by GMS sliding on h-BN, as shown in Fig. 3d. Although the superlow friction between the graphene and h-BN layers has been predicted theoretically^15^, this work presents the experimental measurement of friction between heterogeneous interface of graphene and h-BN layers, where superlow friction is achieved.

## Discussion

The comparison of friction coefficient for different tribo-pairs shows the excellent friction reduction performance of graphene. SiO_2_/SiO_2_ tribo-pair exhibits the highest friction coefficient owing to the strong covalent bond interactions between the SiO_2_ surfaces. The presence of graphene on the substrate can significantly reduce surface dangling bonds at the SiO_2_/SiO_2_ interface, thus leading to a relatively lower friction. It is worth noticing that while the sliding happens between graphene and SiO_2_ for both MLG/SiO_2_ and SiO_2_/G_sub_ tribo-pairs, the friction coefficient of MLG/SiO_2_ is less than half of the SiO_2_/G_sub_. This is at the first glance out of expectation, as the MLG grown on microsphere is rough, polycrystalline as indicated by the SEM, Raman spectra results, while the transferred CVD graphene on the flat substrate is monolayer with large grain size. To figure out this counterintuitive result, Raman spectroscopy measurement of the scanned area on the SiO_2_ substrate after sliding with GMS was conducted (see Supplementary Note 8 and Supplementary Fig. 16). The scanned area is visible in SEM image. G peak and 2D peak which are typical for graphene can be clearly detected in the scanned area, but is undetectable outside the scanned area, suggesting the sliding induced cleavage and transfer of the graphene layer(s) of MLG from the microsphere to the SiO_2_ substrate, resulting in the formation of protective graphene layer(s) on the substrate. Afterwards, the shear plane shifts from graphene/SiO_2_ to between the graphene layers, which accounts for the lower friction. Nevertheless, for SiO_2_ microsphere sliding against monolayer graphene, it is difficult for the monolayer graphene on the substrate to transfer onto the SiO_2_ microsphere unless the graphene is worn out. The comparison of SiO_2_/G_sub_ and MLG/SiO_2_ tribo-pairs proves a relatively smaller friction between the graphene layers, thus the coating of MLG on the SiO_2_ microsphere can offer a better tribological performance. For the MLG/G_sub_ tribo-pair, the graphene coatings on both sides provide a shear plane between graphene layers with weak shear strength, giving rise to the lowest friction of the four tribo-pairs.

The most impressive result of GMS probe is the achievement of superlubricity when sliding against HOPG or nature graphite. This superlow friction can be ascribed to the following reasons: the freshly cleaved HOPG surface is single-crystalline with a grain size from several tens micrometers to several millimeters, and the coating of graphene films on the SiO_2_ microsphere can significantly eliminate the dangling bonds, leaving predominately weak van der Waals forces at the sliding interface, which has been assumed to be an important reason for the lubricity of 2D materials.

**Figure 4:**
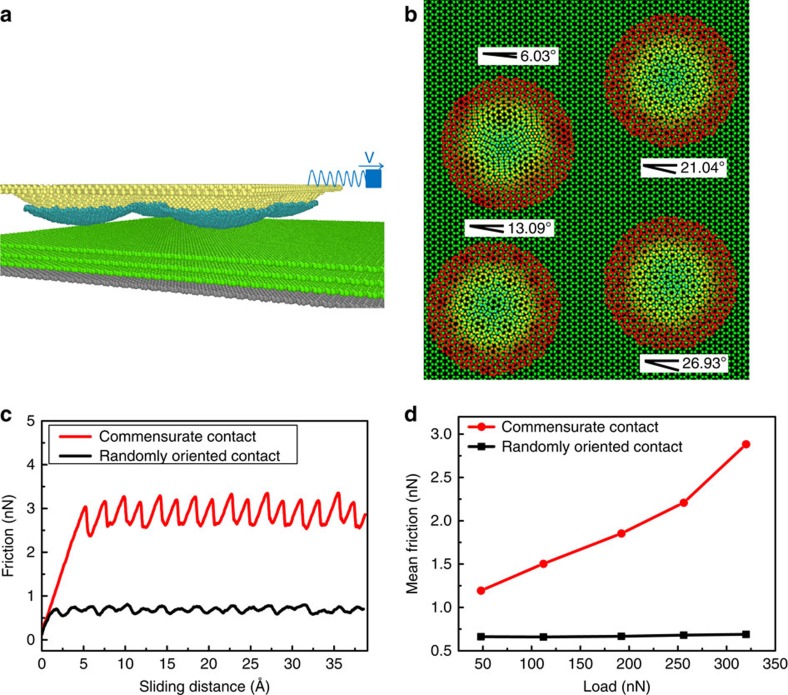
Simulation results of the randomly oriented multi-asperity contact model. (**a**) Side view of the system. The yellow atoms represent the rigid upper base with four diamond bumps. The blue atoms denote the graphene flakes wrapped on the asperities. The green atoms represent the graphite substrate and the bottom grey atoms indicate the fixed layer. (**b**) Top view of the system. The graphene-coated diamond hemispheres represent the asperities on the GMS microsphere. The orientation angles are chosen randomly between the graphene flakes and the underlying graphite substrate. The atoms of the graphene flakes are colour coded based on the Z direction coordinate of these atoms. (**c**) Friction force with the sliding distance of the randomly oriented multi-asperity model in comparison with the commensurate model, with an applied load of 320 nN. (**d**) Friction force with a function of applied load for the two models.

Furthermore, besides interlayer van der Waals interaction, the interfacial atomic registry also plays an important role in superlubricity. Based on the measured mechanical properties and the surface morphology of the microsphere, numerical simulation is conducted to calculate the contact properties between GMS microsphere and HOPG (see Supplementary Note 9 and Supplementary Figs 17–19 for details). Under applied load of 1.4 μN in a case study, five asperities on the rough microsphere surfaces are calculated to be in direct contact with the HOPG surface and together bear the normal loading, as shown in Supplementary Fig. 19. The calculated maximum asperity contact pressure can reach approximately 1 GPa locally. These five asperities are separated from each other by a distance of at least 200 nm, which is larger than the estimated single crystalline grain size on the microsphere. Thus we can deduce a multi-asperity contact mode between the rough microsphere and the flat HOPG, which is consistent with the linear friction laws as described in the Results section. The graphene nanograin on each asperity should orient randomly as compared with the single crystalline HOPG substrate, which may lead to an overall incommensurate state for the asperities. In order to validate this mechanism of superlubricity produced by GMS, MD simulations were conducted by sliding the graphene-wrapped 10-nm-diameter diamond bumps together with a rigid upper base against a single crystalline graphite substrate to imitate the multi-asperity contact in experiments. The schematic contact geometry is shown in Fig. 4a,b. Graphene flakes were wrapped on the asperities with randomly chosen misfit angles (*θ*_1_=6.03°, *θ*_2_=13.09°, *θ*_3_=21.04°, *θ*_4_=26.93° as denoted in Fig. 4b) relative to the substrate lattice orientation, which exhibits different periodicity of the moiré pattern. As a comparison, a separate model with commensurate contact between graphene-wrapped asperities and graphite substrate was established. In this commensurate case, except for the different orientation angle (*θ*_1_=*θ*_2_=*θ*_3_=*θ*_4_=0°) and strict lattice match with the substrate, all the other contact geometries were kept the same for the curved graphene flakes in order to preclude other affecting factors, such as contact area and pressure. As shown in Fig. 4c,d, the distinct stick-slip friction behaviour of atomic lattice periodicity is observed for the commensurate contact, but absent for the randomly orientated graphene flakes, which is in accordance with the disappearance of the stick-slip motion experimentally (see Supplementary Fig. 10). Also the average friction is much larger for the commensurate contact. With the increase of the normal load, the friction force of commensurate system increases linearly, while the friction of randomly orientated graphene system shows no obvious change with normal load. Both the friction and friction coefficient (slope) show large differences for the commensurate and randomly orientated contact. So the graphene nanograins oriented randomly on top of the asperities could lead to an overall incommensurate state under multi-asperity contact conditions. All the asperities can neither synchronously attain commensurate contact, nor trigger the slip process at the same time, which will lead to smooth sliding and minimum energy dissipation. The overall incommensurate geometry and superlubricity have also been verified experimentally: the friction force between MLG and HOPG is independent on the rotation angle between the microsphere and HOPG substrate (see Supplementary Note 10 and Supplementary Fig. 20). Furthermore, the frictional properties of MLG-coated asperity was also studied by a separate MD simulation for comparison, which shows similar behaviours to the monolayer graphene-coated asperity (see Supplementary Note 11 and Supplementary Fig. 21).

The technique of directly coating graphene on SiO_2_ microspheres with curvature is new and could be extended not only to the growth of other 2D materials such as MoS_2_ on microsphere probe, but also to coating of macroscale objects for the sake of friction-reduction and wear-resistance. Another advantage of the present method is the controllable thickness and microstructure of the graphene layers grown on the microsphere, which enables tuning of frictional behaviours in an active manner. This shows attractive prospect in establishing microscale superlubricity by coating graphene or other 2D layers on the sliding surfaces. In addition, the GMS may also possess interesting electronic, optical or even biological properties to be discovered.

## Methods

### Preparation of graphene-coated microsphere probe

SiO_2_ microspheres purchased from Suzhou Nano-micro Technology with mean diameter of 8 μm were used as substrate for CVD growth. Firstly they were directly coated with polycrystalline graphene film by a metal-catalyst-free CVD method, and then attached onto the cantilever. The SiO_2_ powder was supersonic dispersed into ethanol and then dropped on quartz plate followed by being loaded into the center of quartz tube in a CVD furnace. Before heating, 1,000 standard cubic centimeters per minute (sccm) Ar was flushed into the quartz tube to clear air trapped in the system. The furnace was then heated up to the targeted temperature 1,130 °C under a constant pure hydrogen gas flow of 200 sccm. When the furnace was heated to the targeted temperature, the H_2_ gas flow and Ar gas flow were tuned to 50 sccm simultaneously, and a CH_4_ gas flow of 6.3 sccm was introduced into the system for 2 h to initiate graphene growth. After finishing the CVD growth process, the furnace was opened to allow the system cooling rapidly to room temperature.

The preparation of SiO_2_ microsphere probe was carried out under MFP3D SA (Asylum Research, Santa Barbara, CA, USA), with a camera lens and a light path underneath the transparent quartz plate, which is convenient for precisely locating the cantilever and the SiO_2_ microsphere. Graphene-coated microsphere was attached to AFM cantilever (Nanosensor TL-FM) with UV light solidify glue, and then exposed to UV light for 20 min.

### TEM sample preparation of graphene on SiO_2_ microsphere

The TEM cross-sectional sample was prepared by focused ion beam (FIB), picked out from the top of the microsphere. Au film was firstly sputtered on the graphene sample and Pt film was deposited subsequently as protection layers. The deposition of Au and Pt films, aiming to protect the interested region during FIB milling, was under low energy to prevent damage to the lamellar structure of MLG.

### Characterization

The Raman spectra of GMS was collected on Horiba HR800 with a laser wavelength of 514 nm and × 50 objective lens under ambient conditions, and the laser spot size was 1 μm. The morphology of GMS was observed by SEM (FEI Quanta 200 FEG). TEM (JEOL 2011) experiment was conducted to observe the clear lamellar structure of graphene.

### Friction force microscopy tests

The friction tests were conducted by MFP3D SA (Asylum Research) AFM in lateral force mode under ambient atmosphere, at a temperature of 26±2 °C and with a relative humidity of 18±3%. The friction tests under different atmospheres were conducted by NT-MDT AFM with a chamber to control the atmosphere up to 51% relative humidity.

The normal spring constant of the microsphere probe prepared by the tipless cantilever (Nanosensor TL-FM) is 2.5±0.5 N m^−1^. Friction force was measured by the half width of the lateral force loop evaluated for 16 scan lines under each load, and the normal spring constant of the cantilever was calibrated using the thermal noise method. All the experiments presented in Fig. 3 were conducted with the same scan size of 1 μm in reciprocating mode by disabling the slow scan direction, with a scan rate of 2 μm s^−1^. Bulk h-BN (from XFNANO) was used to evaluate friction between heterostructure of graphene and h-BN.

In order to derive the lateral force from the voltage signal, the lateral spring constant of microsphere probe was calibrated using a diamagnetic lateral force calibrator^34^, which is estimated to be 76.64 μN V^−1^ for graphene-coated SiO_2_ microsphere probe and 79.09 μN V^−1^ for bare SiO_2_ microsphere probe.

### Molecular dynamics simulation

The atomistic configuration consisted of one graphite substrate with four graphene layers and altogether 67,488 carbon atoms where the atoms in the bottom layer were kept fixed during the simulation, four graphene wrapped diamond bumps (cutting from diamond nanospheres) to simulate the nanoscale asperities with a diameter of 10 nm and altogether 16,780 carbon atoms, and also one upper base to simulate the bulk microsphere with 10,588 carbon atoms. The size of simulation box is 243 Å × 183 Å × 36.5 Å. The graphene flakes were wrapped on the asperities through Leonard-Jones (LJ) potential to avoid possible wrinkling and excessive strain. The AIREBO potential was used to describe the interatomic interaction within and between the graphene flakes and graphite substrate^35^. The MD simulations were based on the large-scale atomic molecular massively parallel simulator^36^. All the diamond atoms (include the bumps and the upper base) were treated as rigid body and pulled by a virtual atom with a constant velocity of 2 m s^−1^ along *x* direction through a spring with a stiffness of *k*=10 N m^−1^, which was used to mimic the compliance of an AFM system^37^. In addition, a constant normal force in the range of 48∼320 nN was applied on top of the upper base to simulate the normal loading in AFM measurements. Langevin thermostat was applied to the portion of the graphene flakes and the graphite substrate away from the contact area to control the system temperature at 10 K, in order to better demonstrate the intrinsic frictional behaviours without thermal fluctuations. In a separate calculation, the frictional behaviour at 298 K was also studied, acquiring basically the same trend.

### Data availability

The data that support the findings of this study are available from the corresponding authors on request.

## Additional information

**How to cite this article:** Liu, S.-W. *et al*. Robust microscale superlubricity under high contact pressure enabled by graphene-coated microsphere. *Nat. Commun.* **8,** 14029 doi: 10.1038/ncomms14029 (2017).

**Publisher's note:** Springer Nature remains neutral with regard to jurisdictional claims in published maps and institutional affiliations.

## Supplementary Material

Supplementary Information Supplementary Figures, Supplementary Notes, Supplementary Methods and Supplementary References.

Supplementary Movie 1 Supplementary video 1 is the side view of the simulation system showing that a water droplet is repelled away from the contact region when a graphene flake covered asperity (not shown) is loaded on a graphite surface.

Supplementary Movie 2 Supplementary video 2 is the top view of the simulation system showing that a water droplet is repelled away from the contact region when a graphene flake covered asperity (not shown) is loaded on a graphite surface.

Supplementary Movie 3 Supplementary video 3 is the side view of the simulation system describing that a water droplet is split into several smaller droplets and squeezed out of the contact area when a graphene flake covered asperity (not shown) is loaded and sliding on a graphite surface.

Supplementary Movie 4 Supplementary video 4 is the top view of the simulation system describing that a water droplet is split into several smaller droplets and squeezed out of the contact area when a graphene flake covered asperity (not shown) is loaded and sliding on a graphite surface.

Supplementary Movie 5 Supplementary video 5 is the side view of the simulation system showing the sliding process of the asperity surrounded by small water droplet and then encountering a large water droplet.

Supplementary Movie 6 Supplementary video 6 is the top view of the simulation system showing the sliding process of the asperity surrounded by small water droplet and then encountering a large water droplet.
