# Supplementary material for: Robust microscale superlubricity under high contact pressure enabled by graphene-coated microsphere
Source: Nat Commun. 2017 Feb 14;8:14029. doi: 10.1038/ncomms14029 (PMC5316838; doi:10.1038/ncomms14029)
Supplement: Supplementary Information — Supplementary Figures, Supplementary Notes, Supplementary Methods and Supplementary References. [file ncomms14029-s1.pdf]

Supplementary Note 1: Grain size of the graphene grown on SiO_2/Si flat substrate

In order to estimate the grain size, contact-mode AFM measurement of the graphene films on SiO_2 flat substrate is conducted. The graphene on flat SiO_2/Si substrate is grown together with that on the SiO_2 microsphere under the same growth conditions. As can be seen in Supplementary Figure 1, AFM height image and lateral force trace image of the graphene film exhibit randomly-nucleated and rounded-shape graphene islands. The diameters of the islands range from several tens of nanometers to roughly 250 nm with an average of 162 nm. Presumably, each island corresponds to a single crystalline domain. In order to verify the correlation between the size of rounded graphene islands and the single crystalline grain size, atomic-resolution friction images are obtained with the same sample orientation and scanning orientation on each island as shown in Supplementary Figure 2 (filtered to improve contrast) and Supplementary Figure 3 (unfiltered raw images). Clearly, the three images (b), (c) and (d) show totally different honeycomb lattice orientations for different islands. Furthermore, as shown in Supplementary Figure 4 (filtered) and Supplementary Figure 5 (raw) within a certain island, the atomic-resolution friction images for three different areas show the same lattice orientation (within our experimental uncertainty related to a small tip motion hysteresis and sample/tip drift), consistent with the single-crystalline hypothesis of the island.

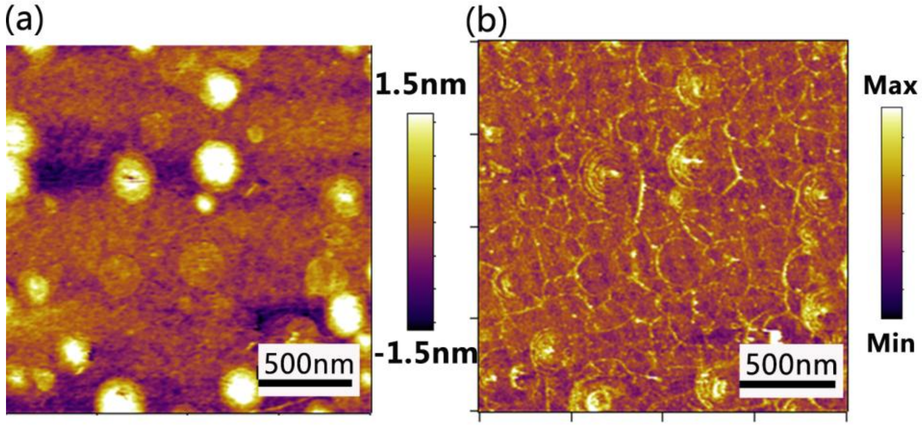

Supplementary Figure 1 | AFM measurement of graphene film grown on flat SiO_2 substrate. (a) Height image. (b) Lateral force trace image of the same scan region.

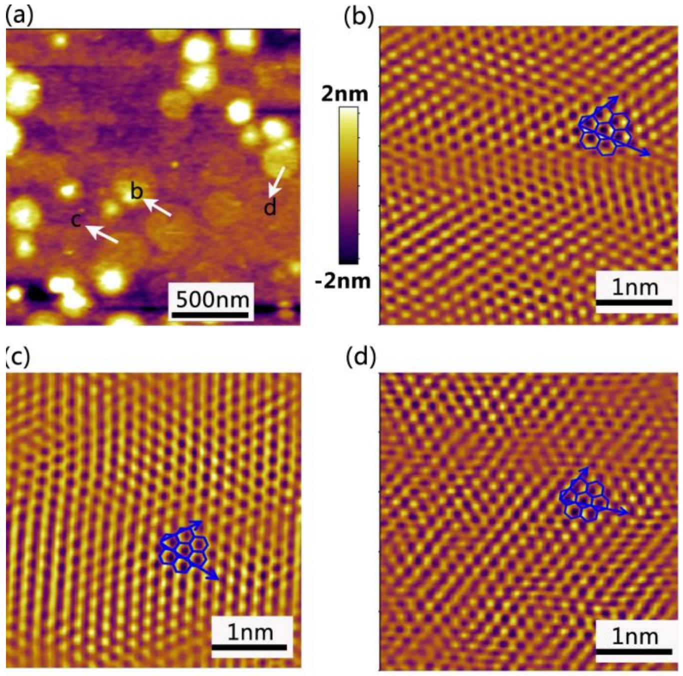

Supplementary Figure 2 | Atomically resolved AFM measurement for grain size estimation of the graphene film grown on flat SiO_2 substrate. (a) Height image. (b, c, d) Filtered atomically resolved lateral force images for three different islands marked by the start of the white arrows in (a).

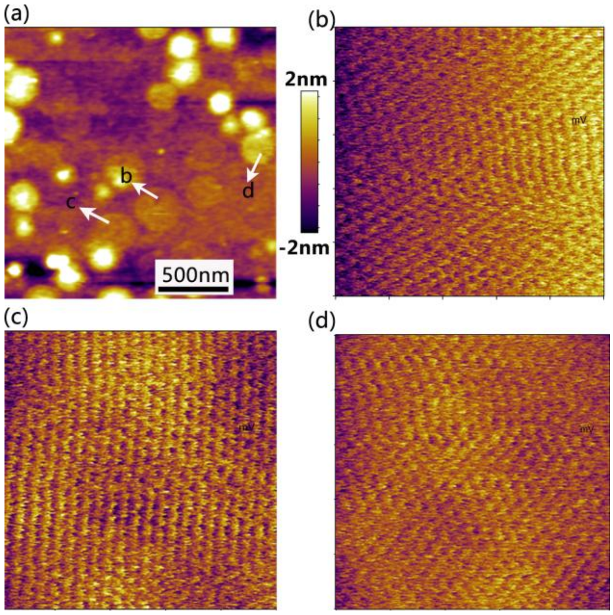

Supplementary Figure 3 | Atomically resolved AFM measurement for grain size estimation of the graphene film grown on flat SiO_2 substrate. (a) Height image. (b, c, d) Raw atomically resolved lateral force images for three different islands marked by the start of the white arrows in (a).

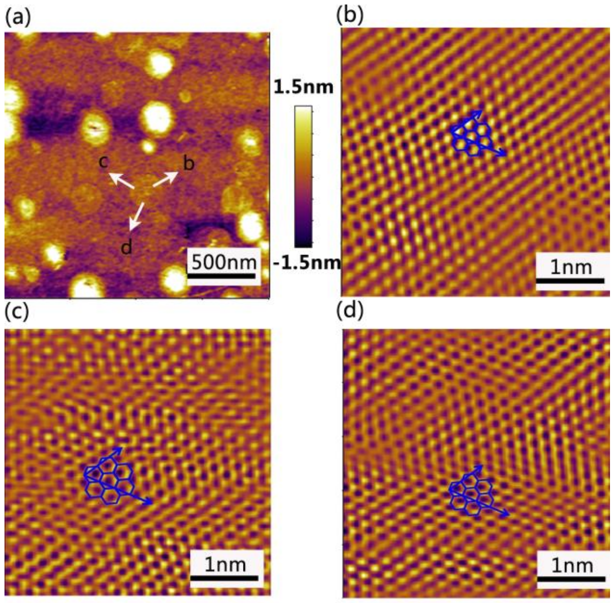

Supplementary Figure 4 | Atomically resolved AFM measurement for grain size

estimation of the graphene film grown on flat SiO_2 substrate. (a) Height image. (b, c, d) Filtered atomically resolved lateral force images for different areas within the same island marked by the start of the white arrows in (a).

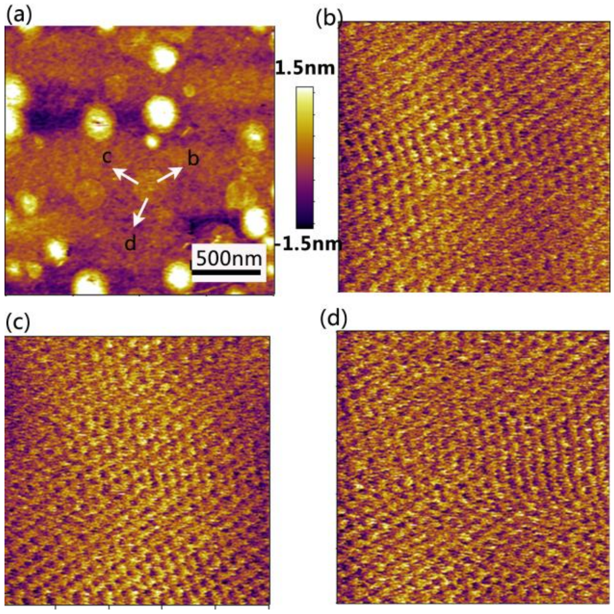

Supplementary Figure 5 | Atomically resolved AFM measurement for grain size estimation of the graphene film grown on flat SiO_2 substrate. (a) Height image. (b, c, d) Raw atomically resolved lateral force images for different areas within the same island marked by the start of the white arrows in (a).

Supplementary Note 2: Adhesion forces measured for the tribo-pairs

The adhesion forces for the four different tribo-pairs are shown in Supplementary Figure 6. Graphene can reduce adhesion force of SiO_2/SiO_2 tribo-pair. The adhesion force contributes to friction at the nanoscale, and explains the initial friction force at zero applied normal load.

For the two tribo-pairs of MLG/HOPG and MLG/G_{sub} when the friction force is relatively low, the friction curve still exhibits linear relationship as shown in Supplementary Figure 7. Most interestingly, friction exists at 0 applied load and even in negative load. The adhesion force between MLG and HOPG is estimated to be 71 nN as shown in the inset of the Supplementary Figure 7a, which is one of the main sources to the offset friction force at 0 applied load and even at negative load.

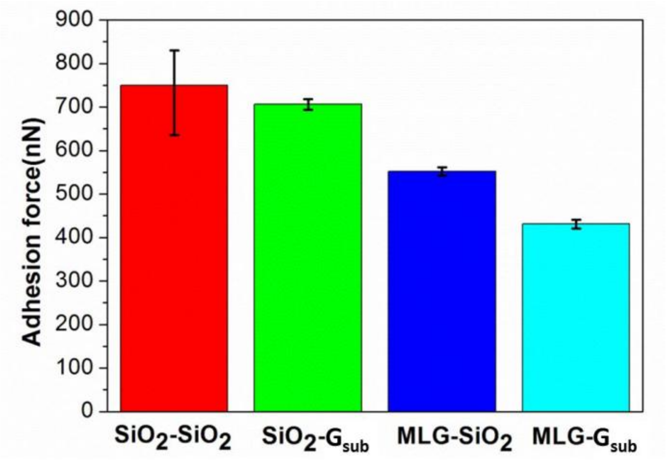

Supplementary Figure 6 | Adhesion forces for the four different tribo-pairs. MLG represents multilayer graphene grown directly on SiO_2 microsphere, and G_{sub} represents transferred CVD grown monolayer graphene. Error bars are defined as s.d.

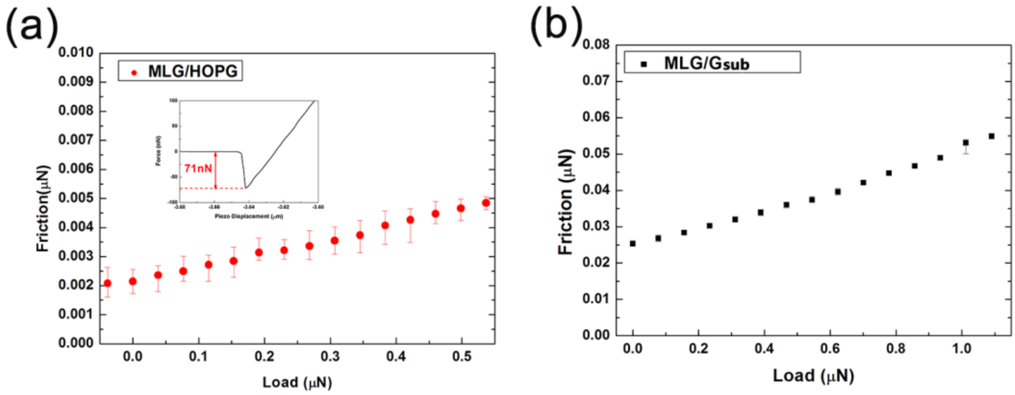

Supplementary Figure 7 | Friction force with applied normal load for the two tribo-pairs of MLG/HOPG (a) and MLG/G$_{sub}$ (b). The inset in (a) presents the pull-off force measured between MLG and HOPG when the graphene coated microsphere is separated from contact with the HOPG surface. Error bars are defined as s.d.

Supplementary Note 3: Different friction coefficients for CVD graphene, mechanical exfoliated graphene and HOPG

Firstly, Friction coefficient between MLG microsphere and mechanically exfoliated monolayer graphene (MEG) on SiO_2 substrate has been measured to be 0.0075 as shown in Supplementary Figure 8. MEG is generally believed to be defect-free, while it is inevitable to introduce defects and contaminations during the growth and transfer process of CVD graphene. So the approximately 3 fold difference in the friction coefficient between MLG/Gsub and MLG/MEG could be attributed to the defects and contaminations in monolayer CVD graphene. Secondly, there exists approximately 2-3 fold difference in the friction coefficient for MLG/MEG and MLG/HOPG. The layer dependence of friction has been reported, where the monolayer MEG shows 2 times higher friction than bulk graphite due to the puckering effect[1]. And this phenomenon is also closely related to the roughness of the underlying substrate of graphene[2]. Besides, the enhancement of friction by atomic roughness has also been studied in previous studies[3]. Here, in order to further compare the friction difference between MLG/MEG and MLG/HOPG, we supplemented additional molecular dynamics simulations to study the sliding friction between graphene coated microsphere and MEG on rough fractal surface of amorphous substrate, as shown in Supplementary Figure 9a. The simulation details are described in the Supplementary Methods Section. The friction curves with respect to the sliding distance of the support are shown in supplementary Figure 9b. For the graphene on rough amorphous surface, dramatic friction fluctuation with a much larger wavelength than atomic-scale

one appears, due to the random rough asperities on the substrate. The mean friction on rough fractal surface is also larger than that on HOPG, which is also in accordance with our experiment results of graphene coated microsphere probe sliding on mechanical exfoliated graphene. To confirm the role of surface roughness in the friction behavior, we also conduct a separate simulation with atomically-smooth surface of the same amorphous materials. The results show that the friction is stable and much smaller than the rough surface. These results show that the surface roughness can significantly influence friction force and explain the friction difference between MLG/MEG and MLG/HOPG in our experiments. By a combined effects of the two possible contributions above, the friction force on CVD graphene could be much larger than on that on HOPG.

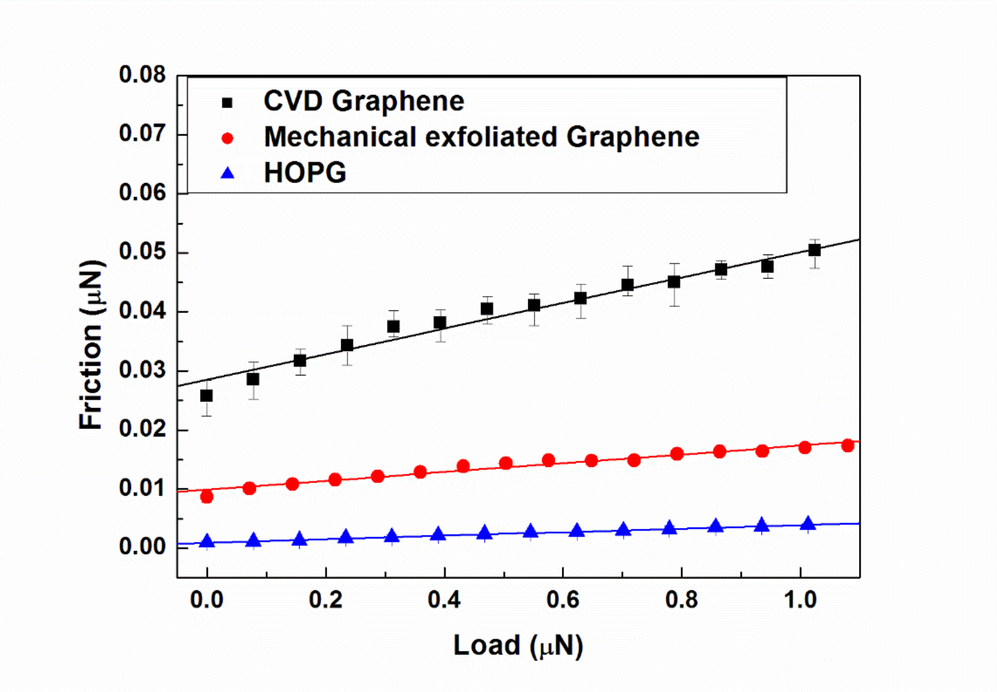

Supplementary Figure 8 | Friction coefficient for graphene coated microsphere against CVD graphene, mechanical exfoliated graphene and HOPG. The slope of

the curve represents the friction coefficient. Error bars are defined as s.d.

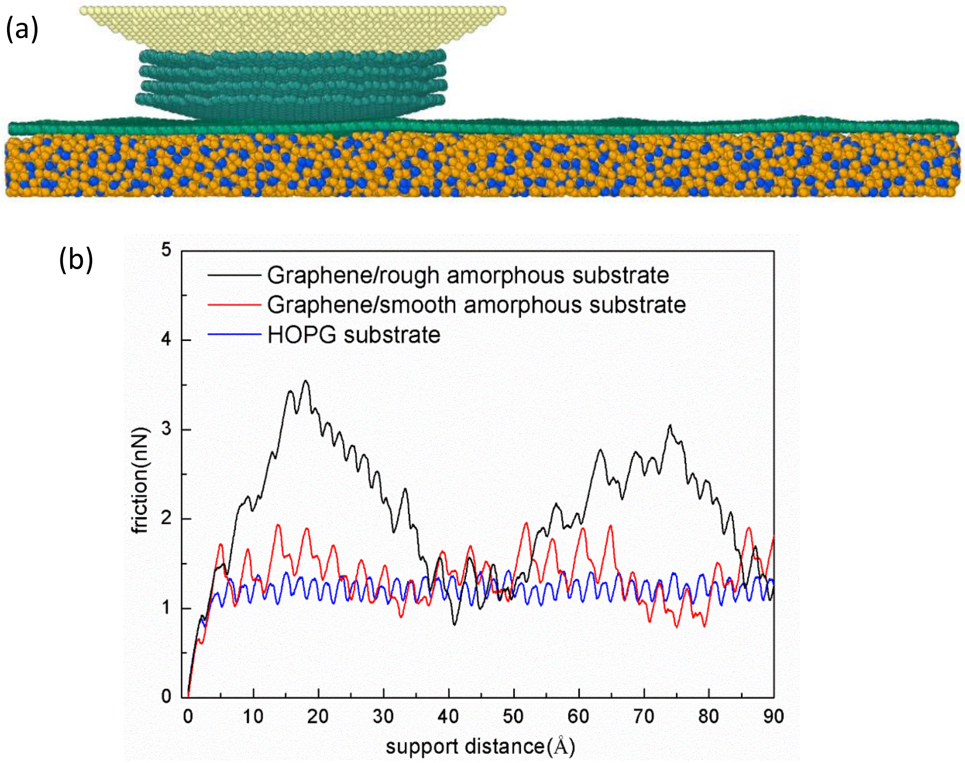

Supplementary Figure 9 | MD simulations of multi-layer graphene on the rough amorphous surface. (a) Side view of the configuration. (b) Effect of different substrates (including graphene on rough amorphous substrate, graphene on smooth amorphous substrate, and HOPG substrate) on friction. Normal load: 40 nN, sliding velocity 2 m s^{-1}.

Supplementary Note 4: Nanoscale friction loop of SiO_2/HOPG and MLG/HOPG tribo-pair

Supplementary Figure 10 presents the friction loop, where the area enclosed by the trace and retrace curves represents the average friction and the frictional energy dissipation at the nanoscale. The SiO_2/HOPG tribopair shows relatively large friction force, however, friction almost vanishes between MLG/HOPG, where superlubricity is obtained at the nanoscale.

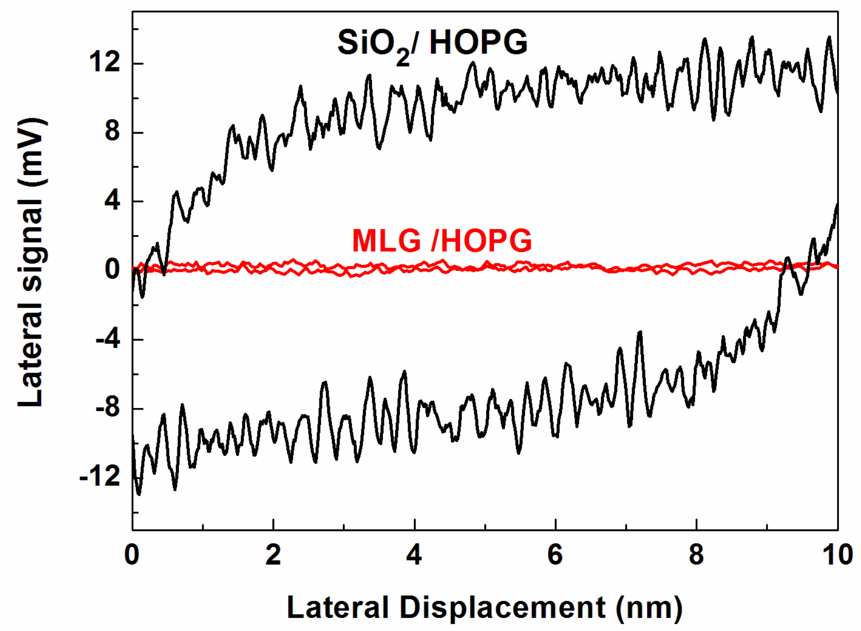

Supplementary Figure 10 | Friction loops of SiO_2/HOPG and MLG/HOPG tribo-pair. The scan size is 10 nm with a normal load of 0.5 μN.

Supplementary Note 5: Post-friction characterization on both the microsphere and the substrate

In order to better characterize the wear mechanism and its relation with diminishing superlubricity after long period of sliding friction, AFM scanning over a relatively large square area (10 μm×10 μm) was adopted with a scan frequency of 3 Hz instead of the single-line reciprocating mode. Supplementary Figure 11a indicates that after nearly 27000 cycles the friction force starts to increase. The wear track of 10 μm ×10 μm on the graphite substrate is measured by AFM contact mode just after the wear test without lifting the probe up, as shown in Supplementary Figure 11b. Otherwise, the wear track with a depth of approximately 1 nm is too shallow to be captured by optical microscopy. Moreover, no clear evidence of structure change and wear on the microsphere is observed by Raman spectra. The gradual increase of friction may be attributed to the mild wear on the graphite substrate side.

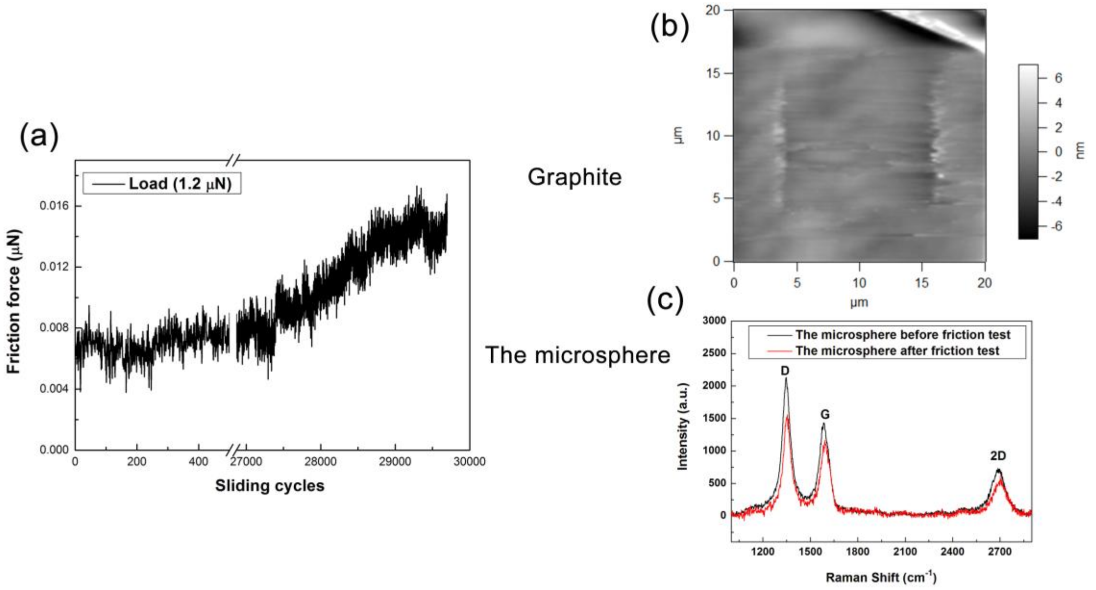

Supplementary Figure 11 |Post-friction characterization on graphite and the microsphere. (a) Time evolution of friction for graphene coated microsphere on

graphite under applied load of 1.2 μN. Scan size 10 μm × 10 μm, scan rate 3 Hz. (b) The morphology of the scanning area on graphite after friction test. (c) Raman spectra of the graphene coated microsphere before and after friction test.

Supplementary Note 6: Robust superlubricity under different experimental conditions

In order to demonstrate the robustness of this superlubricity, experiments under various severe conditions on graphite have been done, such as the change of relative humidity, scan rate and scan size. As shown in Supplementary Figure 12a, under dry nitrogen or higher relative humidity of 44% as measured by an NT-MDT AFM with environmental chamber, superlubricity is preserved at the same level. Supplementary Figure 12b and 12c indicates that the friction coefficient is not changed with the scan rate and scan size under ambient humidity of 20%. Supplementary Figure 12d shows the lateral force loop with a scan size of 10 μm under a normal load of 1.2 μN. The friction force is reduced to about 1/14 by the coating of graphene on the SiO_2 microsphere, as compared with bare SiO_2 microsphere under the same applied load.

The humidity influence under larger scan size on friction was also investigated. As shown in Supplementary Figure 12e, under the same humidity of 31.8%, the friction force of 10 μm approximate that of 1 μm, with a friction coefficient of 0.003. Even under 51% RH, superlubricity is still maintained although the friction coefficient increases a little bit to 0.006. This humidity independence could be ascribed to the suppressed capillary formation for hydrophobic surface.[4-7] In previous experiments, the sliding friction between the sharp AFM tip and the sample is measured to be independent on the humidity, where either the tip or the sample is made hydrophobic. For the graphene coated probe and graphite tribo-pair used here, however, both sides of the sliding interface are hydrophobic, resulting in humidity independent superlow

friction with multi-asperity contact at a micrometer scale. The water droplet may be squeezed out of the contact area between hydrophobic surfaces, inhibiting the formation of capillary shear force, as will be further explained in Supplementary Note 7.

In summary, superlubricity can be maintained with a scan rate as high as 100 μm s^{-1}, a scan size as large as 50 μm (Supplementary Figure 12f), and relative humidity up to 51% RH, indicating the robustness of superlubricity for graphene coated microsphere.

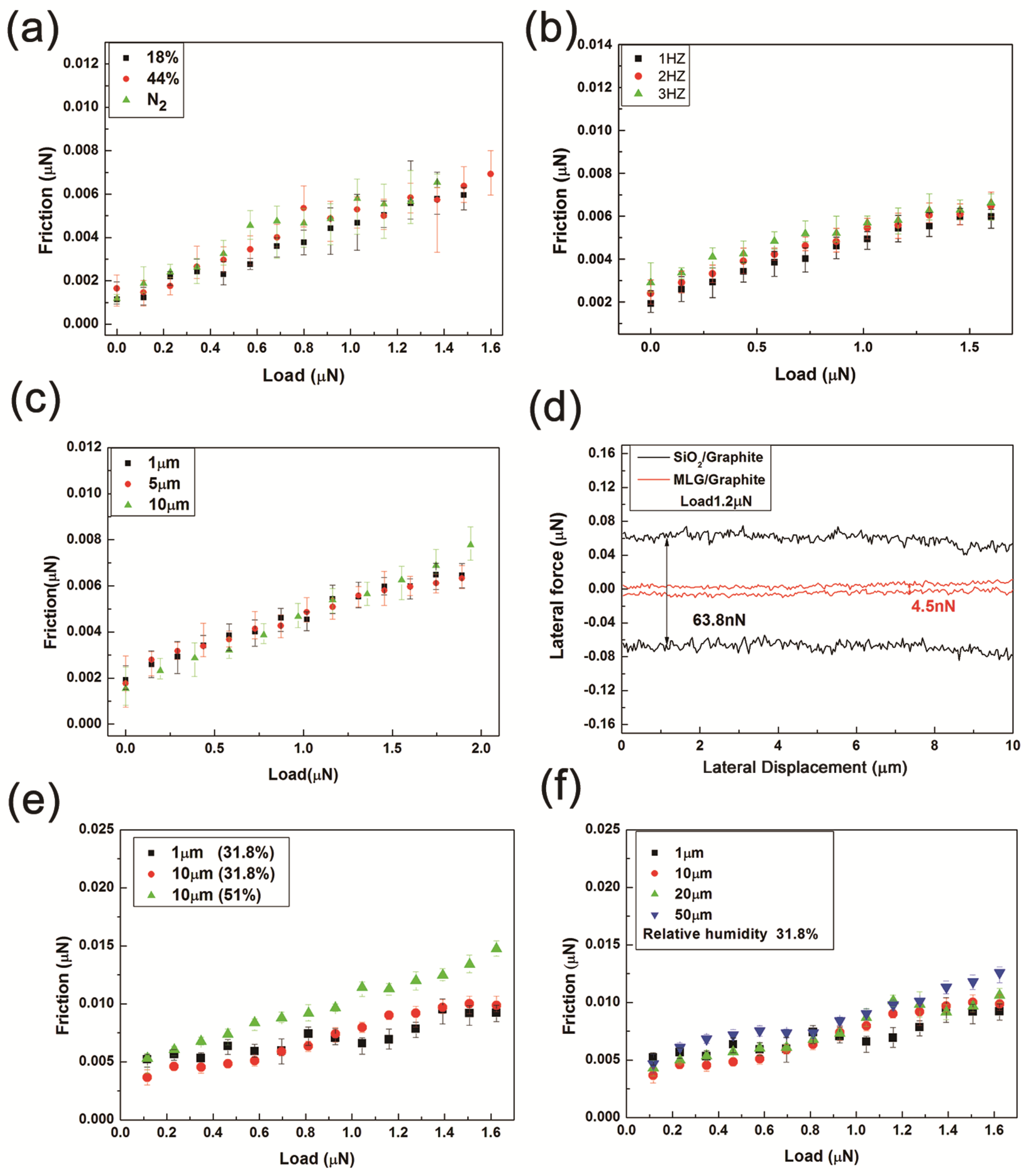

Supplementary Figure 12 | Superlubricity of graphene-coated microsphere on graphite under different experimental conditions. (a) Friction force as a function of applied load under dry nitrogen and 44% relative humidity. Scan size 1 μm, scan rate 1 Hz. (b) Friction force under different applied loads with the change of scan rate. Scan size 5 μm, relative humidity 20%. (c) Friction force as a function of applied load under different scan sizes. Scan rate 1 Hz, relative humidity 20%. (d) Lateral force loop of bare SiO_2 microsphere on graphite and that of MLG on graphite with a scan

size of 10 μm, and under a normal load of 1.2 μN. (e) Friction force measurement with larger scan size under higher humidity. Scan rate 1Hz. (f) Comparison of the friction coefficient under different scan sizes. Relative humidity: 31.8%, scan rate 1 Hz. Error bars are defined as s.d.

Supplementary Note 7: Molecular dynamics simulations on the humidity influence of friction

In the case for hydrophilic surface, water meniscus formed in the contact area results in the formation of capillary shear force, thus greatly influence the friction when sliding. In contrast, the bad wettability on hydrophobic surface is crucial for the humidity-insensitive friction. Water droplet formed on hydrophobic surfaces with a contact angle of about 90° has been reported for graphite surfaces.[8] It is reasonable to assume that water molecules will be squeezed out of the contact area between hydrophobic surfaces during loading and sliding, as a result, the capillary meniscus formation and the bridge-mediated shear force is greatly suppressed for hydrophobic surfaces. MD simulations were conducted to verify the above postulations. As shown in Supplementary Figure 13, during the loading process between a graphene wrapped asperity (not shown) and the graphite substrate, some water droplets formed on the hydrophobic graphite substrate could be repelled away from the contact region (Supplementary Figure 13b), while some water droplets could be split into several smaller droplets, which are inaccessible to the contact area due to the hydrophobic nature of the interface and strong cohesive interaction between the water molecules (Supplementary Figure 13a). As a result, the graphene flakes on asperity are still in direct contact with the graphite substrate, which demonstrates the almost invariable superlow friction in humid as well as in dry environment.

As the case in relatively higher humidity, the friction force shows slightly increase under large scan area (Supplementary Figure 12e). With the increasing scan size, there

are greater odds for neighboring water droplets joining together under the sweeping of the asperities. The coalescence of the water droplets into a relatively larger water droplet in the sliding path will slightly block the motion of the asperities. As shown by the MD simulation in Supplementary Figure 14, the friction force of the asperity surrounded by the small water clusters remains basically unchanged as compared to the dry sliding case in the absence of water molecules, which is in accordance with the experimental results in relatively low humidity. Nevertheless, when the asperity comes across a relatively large water droplet, the friction slightly increases from 0.41 nN to 0.64 nN, which is in accordance with the experimental results under 51% RH with a scan size of 10 μm. However, the capillary formation and capillary shear force are still suppressed due to the hydrophobic nature of the sliding surfaces, so obvious increase in friction is not observed in this case. Friction here is still determined predominantly by the solid-solid direct contact.

In addition, ReaxFF force field is also adopted to describe the interaction between water and graphene,[9] with a defective graphene sheet wrapped on a cylinder in contact with graphite. As shown in supplementary Figure 15, with the introduction of defects and ReaxFF force field, we still find most of the water atoms to be repelled from contact area when loading, and no water molecules re-enter the contact area during the sliding process, which is qualitatively consistent with the TIP4P model above. Only a few water molecules are confined within the defect, which is not sufficient to significantly influence the friction. To this end, we proposed a possible mechanism to account for the experimentally observed stable superlubricity in a certain range of

relative humidity.

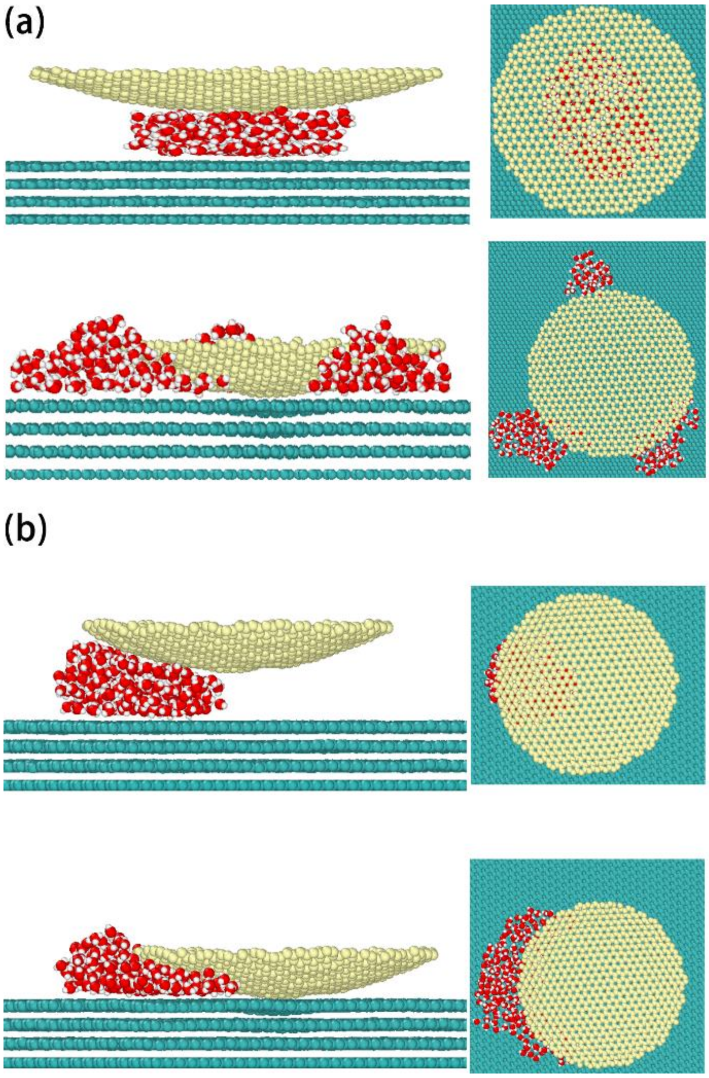

Supplementary Figure 13 | MD simulations of the repulsion of water droplet from the contact area when a graphene flake covered asperity (not shown) is loaded and sliding on a graphite surface. Both side view and top view of the configuration before and after loading/sliding process of the asperity: (a) a water droplet is split into several smaller droplets; (b) a water droplet is repelled away from the contact region.

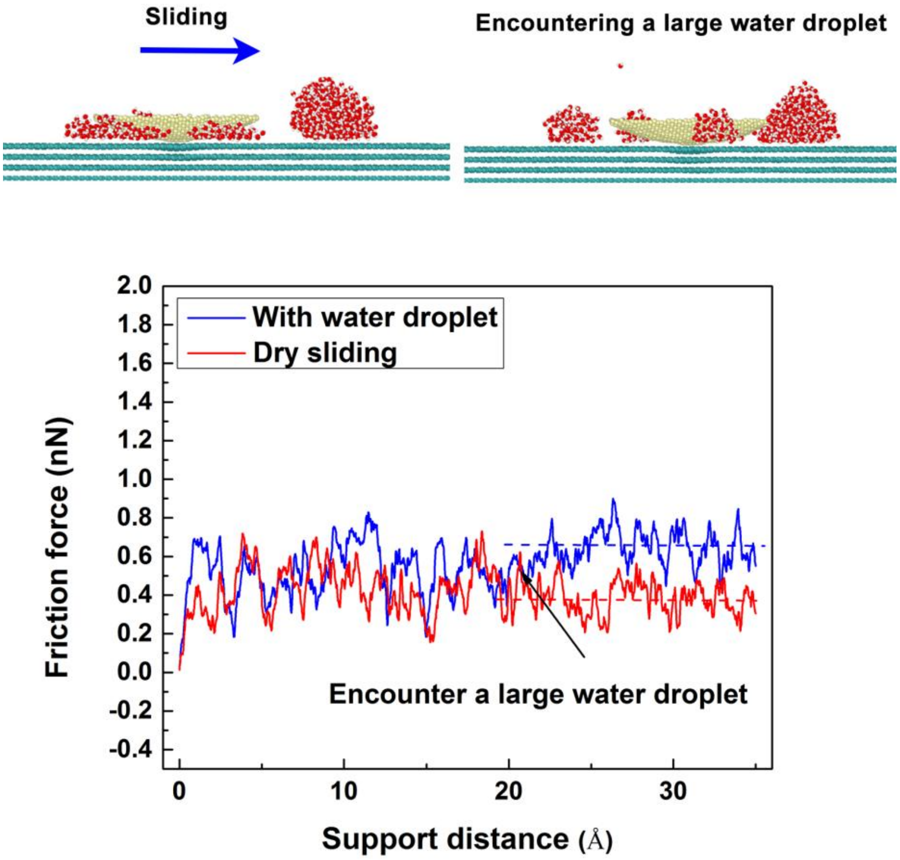

Supplementary Figure 14 | Simulation results of the comparison between dry sliding and the presence of water droplet. Applied normal load 4.8 nN, temperature 298K.

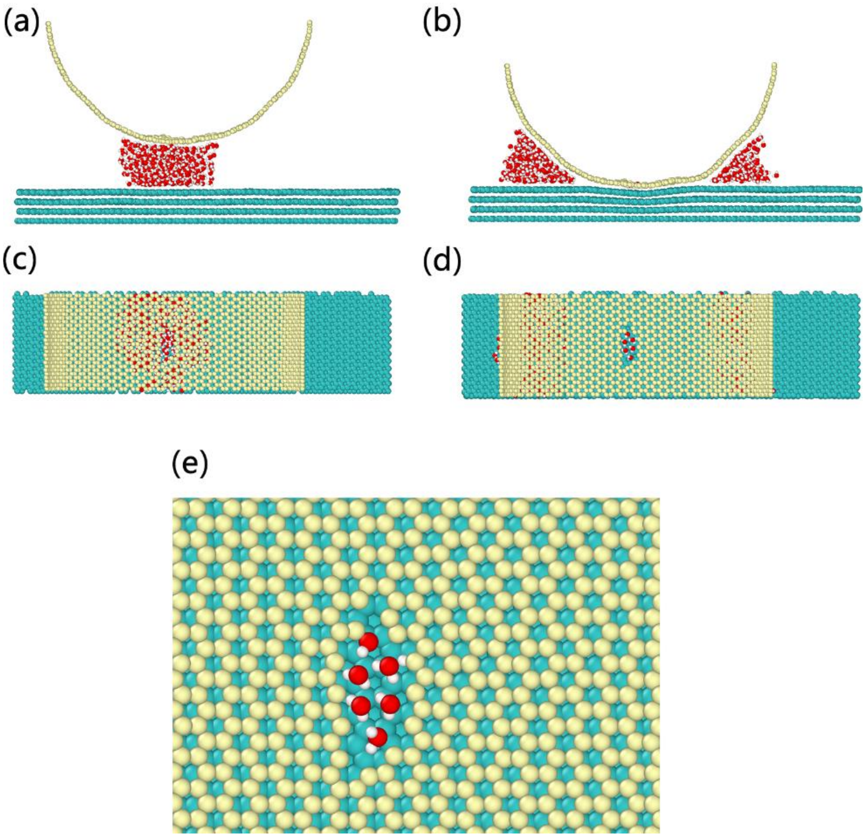

Supplementary Figure 15 | MD simulations using ReaxFF to show the repulsion of water droplet from the contact area when a graphene flake covered asperity (not shown) is loaded and sliding on a graphite surface. Side view (a) (b) and top view (c) (d) of the configuration before and after loading/sliding process of the asperity. (e) Local enlargement of the defects with water molecules, showing the dissociation of a water molecule and interaction with the edge atoms at the defect. Yellow balls represent carbon atoms of the graphene film coated on the upper microsphere, the green ones stands for the carbon atoms of graphite. The red balls are oxygen atoms and the white ones are hydrogen atoms.

Supplementary Note 8: Characterization of the graphene transfer during scanning on SiO_2 substrate

Supplementary Figure 16 shows the SEM image and Raman spectra of the scanning area on SiO_2 substrate. The shear induced cleavage and transfer of graphene from the multilayer graphene coated microsphere to SiO_2 substrate is observed. Raman spectra of the scanning area provide clear evidence of the graphene transfer. The G, 2D and D peaks are detected at about 1580, 2700 and 1350 cm^{-1}, respectively. The darker area in the SEM image indicates that graphene covers the scanning area. The sliding should happen between the graphene layers on the microsphere and the transferred graphene layers on substrate, which explains the lower friction in the MLG/SiO_2 case than that in SiO_2/G_{sub} case.

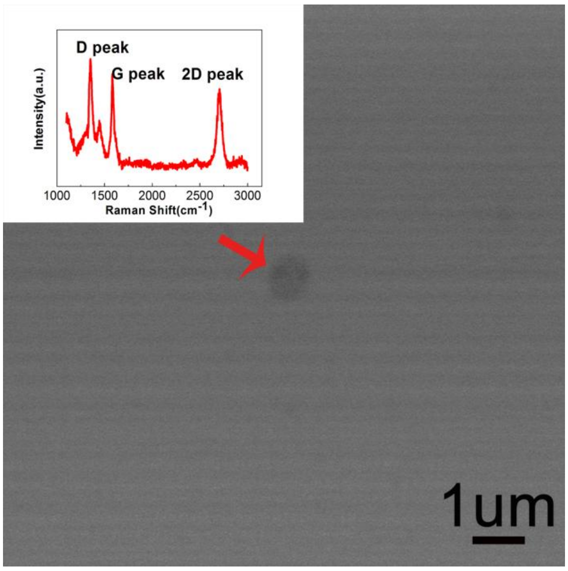

Supplementary Figure 16 | SEM image and Raman spectra of the transferred graphene onto SiO_2 substrate. The scanning area is manifested by the dark area on the SiO_2 substrate with a scan size of 1 μm×1 μm of the MLG/SiO_2 tribo-pair. The inset is the Raman spectra of the scanning area.

Supplementary Note 9: Estimation of the contact properties

In order to calculate the contact area and pressure, direct measurement of Young's modulus and roughness of both bare SiO_2 and graphene-coated microspheres were supplemented. Firstly a nano-indentation test based on AFM is conducted to measure Young's modulus of the microspheres, stemming from a standard routine as reported previously[10,11]. Supplementary Figure 17a schematically illustrates the experimental process. More details are described in the Supplementary Methods section.

The Young's modulus is estimated to be around an average of 20 GPa for graphene coated microsphere and 16.5 GPa for SiO_2 microsphere with relatively good reproducibility. The modulus of SiO_2 microsphere is smaller than 72.9 GPa for bulk fused silica,[12] ascribing to the pores and defects existing in the microsphere.[13] The results show that Young's modulus of the graphene-coated microsphere is slightly larger than the SiO_2 microsphere, probably because the coating of graphene strengthens the defects and the pores, leading to a relatively larger Young's modulus.[14]

The surface roughness of the graphene coated microsphere and bare SiO_2 microsphere is also measured by AFM tapping mode, as shown in Supplementary Figure 18. Both the surfaces are much rougher than their flat counterparts. The standard deviation σ of height distribution is 21.75 nm for graphene coated microsphere and 22.7 nm for SiO_2 microsphere in $1\times1\ \mu m^2$ area. We found that the morphology of graphene films largely follow the underlying SiO_2 microsphere and is slightly smoother than the later one.

Under applied load of 1.4 μN, five asperities on the rough microsphere surfaces are

found to be in direct contact with the HOPG surface and together bear the normal loading, as shown in Supplementary Figure 19. The calculated real contact area is 1.9×10^{-3} μm^2. These contacting asperities are separated from each other by a distance of at least 200 nm, which is larger than the estimated single crystalline grain size.

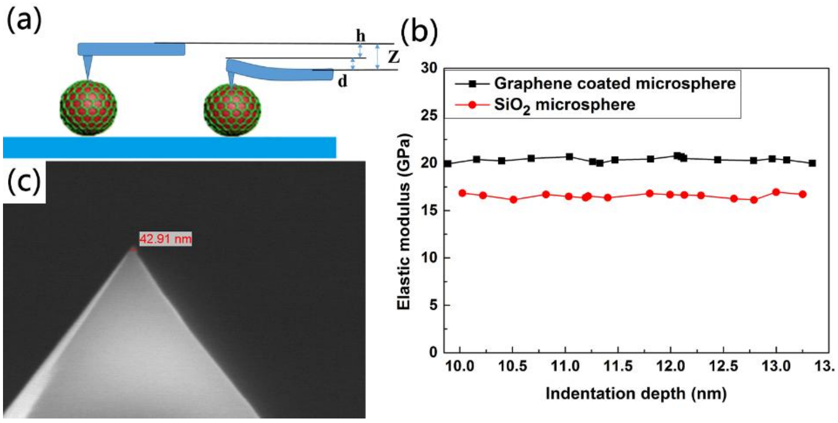

Supplementary Figure 17 | The measurement of Young's modulus of the microsphere. (a) The schematic of the movement of tip-microsphere system under nanoindentation, *h* represents the indentation depth, *d* is deformation of the tip, and *Z* is the downward distance of the cantilever. (b) The estimated Young's modulus with the indentation depth for graphene coated microsphere and SiO_2 microsphere. (c) Measurement of the radius of curvature of the diamond tip used for nanoindentation by SEM image.

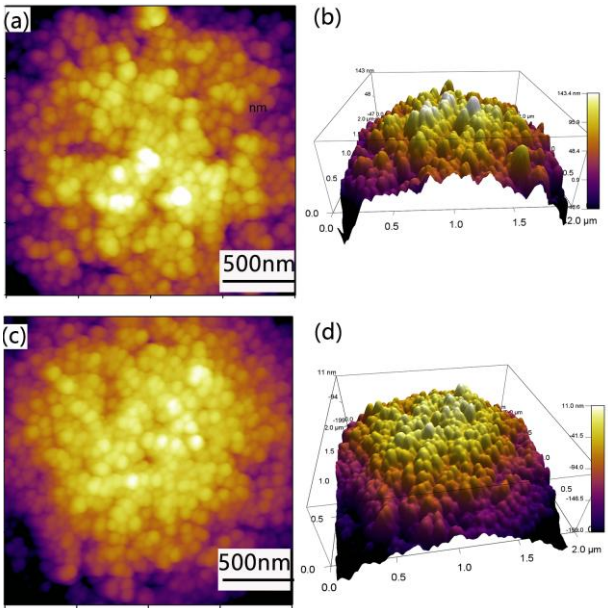

Supplementary Figure 18 | Measurement of the morphology of microsphere. (a, b) AFM tapping mode of height image of SiO_2 microsphere, (c, d) AFM tapping mode of height image of graphene coated microsphere.

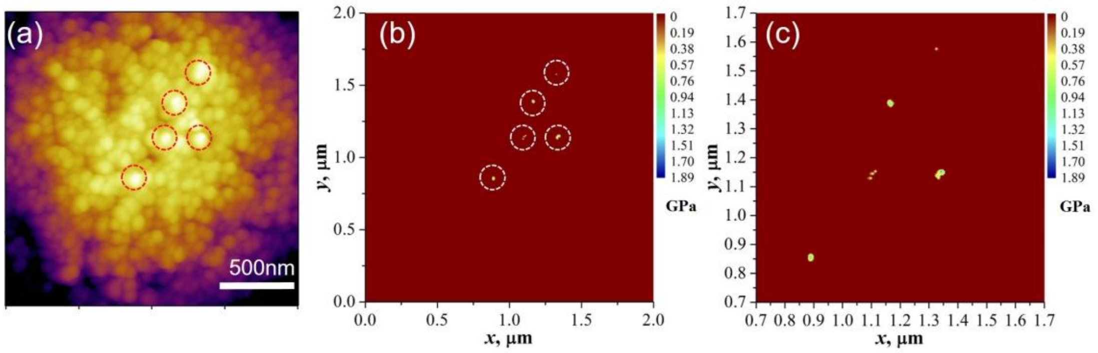

Supplementary Figure 19 | Numeric calculation for a multi-asperity contact (a) surface morphology of graphene coated microsphere; (b) contact pressure corresponding to (a); (c) enlargement of (b) to better illustration of the pressure at contact asperities.

Supplementary Note 10: Effect of rotation angle on friction

The rotation angle between the MLG on the microsphere and HOPG is varied by rotating the HOPG sample with Veeco AFM (Dimension V). The friction force versus rotation angle is shown in Supplementary Figure 20. The applied normal load is 1.44 μN. The friction force with an approximate mean value of 10 nN shows no dependence on the rotation angle.

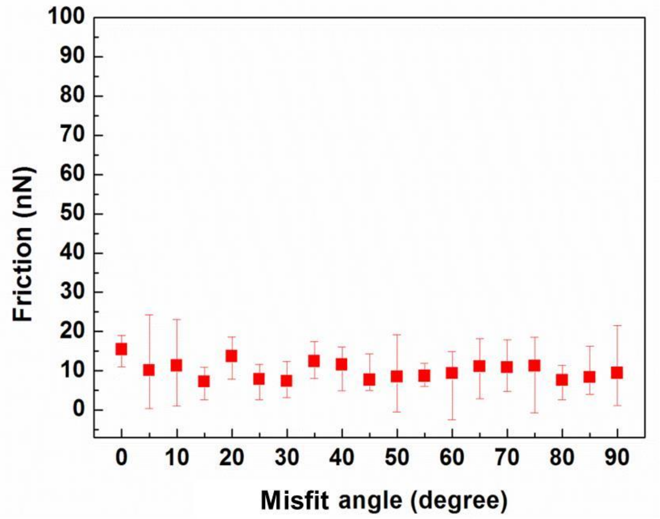

Supplementary Figure 20 | Friction force versus rotation angle between MLG and HOPG. The zero rotation angle is only for reference. Error bars are defined as s.d.

Supplementary Note 11: MD simulation of multilayer graphene coated bump sliding on HOPG

For the multi-layer graphene coated on the bump sliding over HOPG substrate, the interfacial commensurability plays an important role in atomic scale friction. When the bottom graphene layer on the bump and top graphene layer on the substrate is in registry geometrically, the average friction is much larger with obvious atomic-scale stick-slip motion, as shown in Supplementary Figure 21. In contrast, a smooth sliding with smaller average friction is observed for incommensurate contact. This is qualitatively consistent with the results in main text where single layer graphene is wrapped on the bump.

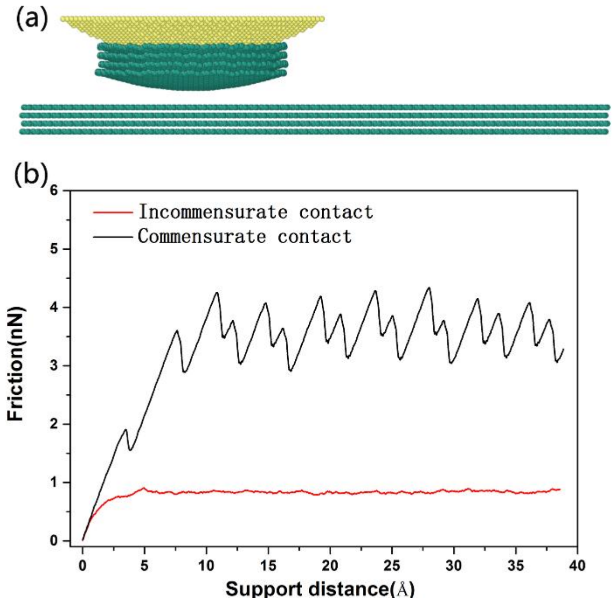

Supplementary Figure 21 | Frictional properties of multilayer graphene coated asperity. (a) Molecular dynamics model of multi-layer graphene sliding against graphite. (b) Comparison between commensurate and incommensurate contact. Normal load: 320 nN, sliding velocity 2 m/s.

Supplementary methods

Molecular dynamics simulation for multilayer graphene on rough amorphous substrate and HOPG. HOPG was modeled by stacking graphene layers by using AIREBO potential, whereas G_{sub} was modeled by single layer graphene covering on an amorphous substrate. The two component types of atoms in the amorphous substrate interact with a Lennard-Jones (LJ) potential. The interactions between the substrate atoms and graphene carbon atoms were simulated using LJ potential with the parameter $\varepsilon_{sub1\text{-}C}$ =0.332 kJ mol^{-1} and $\sigma_{sub1\text{-}C}$ =3.001 Å, $\varepsilon_{sub2\text{-}C}$ =0.332 kJ mol^{-1} and $\sigma_{sub2\text{-}C}$ =3.001 Å.[15] The amorphous substrate was modeled with fractal surface constructed according to the method described previously,[16] with a RMS roughness of 1.65 Å, so that the roughness of monolayer graphene on SiO_2 substrate is similar to previous experimental measurements.[17,18] Langevin thermostat was employed to control the system temperature in these models. The method for applying the load and sliding motion is similar to that described in the main text.

Molecular dynamics simulation for friction in humid environment. To simulate a humid environment, two water droplets (one containing 216 water molecules, another 343 water molecules) were inserted between the graphene wrapped diamond bumps and graphite substrate with the TIP4P model describing the interactions between water molecules.[19] Leonard-Jones (LJ) potential with a long-range Coulombic solver was applied to describe the interaction between water molecules. Interactions between carbon atoms and water molecules were also described by using the LJ potential. The

LJ interaction parameters for the carbon atoms and oxygen atom in water were $\varepsilon = 0.3126\,\text{kJ mol}^{-1}$ and $\sigma = 3.19$ Å, as was used in previous studies.[20] The asperity was dragged by a virtual atom with a constant velocity of 5 m/s through a spring along the x-direction. The normal load was fixed as 4.8 nN, and the temperature was fixed at 298 K in all these simulations.

Molecular dynamic simulation for ReaxFF force field. A cylinder was used for the upper bump here in order to significantly save the computational cost, because ReaxFF simulation is relatively time consuming as compared to classical potentials. A spherical bump was also tested which leads to qualitatively the same conclusion. The simulation cell of the model is 125.7 Å × 32.5 Å, which contains 13056 atoms (6032 atoms for graphite substrate, 343 water molecules in the droplet and 5995 for the asperity). All simulations have been performed with a time step of 0.25 fs. Berendsen thermostat was applied to control the system temperature at 300 K. Constant normal load of 48 nN was applied on the upper bump, and then friction was initiated with a sliding velocity of 5 m s^{-1}.

Nano-indentation experiment on Young's modulus of the microsphere

A diamond tip with the apex diameter of about 42.9 nm (measured from SEM image in Supplementary Figure 17c) and high stiffness of 59.34 nN/nm was used for the AFM nanoindentation test. We used the Hertz elastic contact theory to transfer the obtained force-distance curve to the Young's modulus of the microsphere. The

combined Young’s modulus of tip and the microsphere can be calculated from:

$$h = \left(\frac{9F^2}{16R^* E^{*2}} \right)^{1/3} \qquad (1)$$

where h is the indentation depth and F is indentation force, R^* is the relative radius of curvature and E^* is the combined Young’s modulus of diamond tip and microsphere. Since the parameters of the diamond tip is already known, the Young’s modulus of the microsphere can be calculated, as shown in Supplementary Figure 17b.

Numerical simulation of the contact area and contact pressure

Based on the previously tested parameters of the Young’s modulus and surface morphology, a semi-analytical method and minimization of complementary energy were employed to solve the 3D multi-asperity contact problem under the assumption of elastic deformation for simplicity. The numerical simulation was conducted by establishing a contact model between a realistic rough surface obtained directly from AFM measurement of the graphene microsphere and an ideal plane representing the HOPG surface (Young’s modulus of 17 GPa and Possion’s ratio of 0.3). The conjugate gradient method (CGM) and FFT algorithm were applied to improve computational efficiency. More details of the method are in a previous article.[21]

Supplementary References

1. Lee, C. Li, Q. *et al*. Frictional characteristics of atomically thin sheets. *Science*. **328**, 76 (2010).
2. Cho, D. H. Wang, L. *et al*. Effect of surface morphology on friction of graphene on various substrates. *Nanoscale*. **5**, 3063 (2013)
3. Dong, Y., Wu, X., Martini, A. Atomic roughness enhanced friction on hydrogenated graphene. *Nanotechnology*. **24**, 375701 (2013).
4. Binggeli, M. *et al.* Influence of capillary condensation of water on nanotribology studied by force microscopy. *Appl. Phys. Lett*. **65**, 415 (1994).
5. Sirghi, L. Effect of capillary-condensed water on the dynamic friction force at nanoasperity contacts. *Appl. Phys. Lett.* **82**, 3755 (2003).
6. Ohmae, N. Humidity effects on tribology of advanced carbon materials. *Tribol. Int.* **39**, 1497 (2006).
7. Lee, M. *et al.* Noncontact friction via capillary shear interaction at nanoscale. *Nat. Commun*. **6**, 7359 (2015).
8. Rafiee. J. *et al.* Wetting transparency of graphene. *Nature Mater.* **11**, 217(2012).
9. Rahaman, O., van Duin, A. C. *et al*. Development of a ReaxFF reactive force field for glycine and application to solvent effect and tautomerization. *J. Phys. Chem. B*. **115**, 249 (2010).
10. Tan, S., Sherman, R. L. *et al*. Nanoscale compression of polymer microspheres by atomic force microscopy. Langmuir. **20**, 7015 (2004).
11. Guo, D., Li, J., Xie, G. *et al*. Elastic properties of polystyrene nanospheres evaluated with atomic force microscopy: size effect and error analysis. *Langmuir*, **30**, 7206 (2014).
12. Comte, C., Von Stebut, J. Microprobe-type measurement of Young's modulus and Poisson coefficient by means of depth sensing indentation and acoustic microscopy. *Surf. Coat. Technol*. **154**, 42 (2002).
13. Lim, H. S., Kuok, M. H., Ng, S. C. & Wang, Z. K. Brillouin observation of bulk and confined acoustic waves in silica microspheres. *Appl. Phys. Letters*. **84**, 4182 (2004).
14. Kim, K. H. *et al*. Graphene coating makes carbon nanotube aerogels superelastic and resistant to fatigue. *Nature. Nanotech.* **7**, 562 (2012).
15. Rappé, A. K. *et al.* A full periodic table force field for molecular mechanics and molecular dynamics simulations. *J. Am. Chem. Soc.* **114**, 10024 (1992).
16. Ausloos, M., Berman, D. H. A multivariate Weierstrass-Mandelbrot function. *P. Roy. Soc. A-Math. Phy.* **400**, 331 (1985).
17. Lui, C. H. et al. Ultraflat graphene. *Nature.* **462**, 339 (2009).
18. Zhang, Y., Brar, V. W., Wang, F. *et al.* Giant phonon-induced conductance in scanning tunnelling spectroscopy of gate-tunable graphene. *Nature. Phys*. **4**, 627 (2008).
19. Abascal, J. L. F. Vega, C. A general purpose model for the condensed phases of water: TIP4P/2005. *J. Chem. Phys.* **123**, 234505 (2005).
20. Walther, J. H. Jaffe, R. Halicioglu, T. *et al*. Carbon nanotubes in water: structural characteristics and energetics. *J. Phys. Chem. B.* **105**, 9980 (2001).
21. Zhang, S., Wang, W. *et al.* The effect of surface roughness characteristics on the elastic–plastic contact performance. *Tribol. Int.* **79**, 59 (2014).
